# Smoking cessation interventions on health-care workers: a systematic review and meta-analysis

**DOI:** 10.7717/peerj.9396

**Published:** 2020-06-16

**Authors:** Giuseppe La Torre, Generosa Tiberio, Alessandro Sindoni, Barbara Dorelli, Vittoria Cammalleri

**Affiliations:** Department of Public Health and Infectious Diseases, Sapienza University, Rome, Italy

**Keywords:** Smoking cessation, Interventions, Health-care workers, Systematic review, Meta-analysis

## Abstract

**Objective:**

The authors carried out a systematic review and a meta-analysis on smoking cessation interventions on health -care workers to clarify the state of the art interventions and to identify the best one.

**Materials and Methods:**

This review was registered with PROSPERO: CRD42019130117. The databases PubMed, Scopus, Web of Science and CINAHL were searched until December 2018. Quality of all studies included in the systematic review was assessed according to the Newcastle-Ottawa Scale (NOS) on cohort or cross-sectional studies and to the Cochrane Risk of Bias Tool for Randomized Controlled Trials. Meta-analysis and meta-regression analyses were also carried out for cohort studies (quasi-experimental or a before-after studies design) and clinical trials.

**Results:**

Twenty–four studies have been included in the analysis: four before-after, 13 cross-sectional, three quasi-experimental studies and four clinical trials. Articles were heterogeneous (*P* for homogeneity <0.01), but they have all shown positive results since they reached the goal of smoking cessation among health-care workers, even if with different proportions. Meta-analysis was performed on 10 studies (six cohort studies and four clinical trials), showing a 21% of success rate from the application of smoking cessation interventions, either pharmacological or behavioral ones. The resulted pooled RR (Risk Ratio) was 1.21 (95% CI [1.06–1.38]), being 24% of success rate from clinical trials (pooled RR 1.244; 95% CI [1.099–1.407]) and 19% of success rate from cohort studies (pooled RR 1.192; 0.996–1.426). However, two studies have confidence intervals which include unity and one study has a wide confidence interval; as a consequence, the meta-analysis for its results depends heavily on one single study. Meta-regression analysis revealed that results were influenced by the number of participants.

**Conclusion:**

Both policy and pharmaceutical interventions can obtain positive results in quitting smoking among health-care workers. However, as shown by our review, combination approaches can produce better results in terms of cessation percentages and smoking abstinence.

## Introduction

The World Health Organization (WHO) has defined tobacco use as “the epidemic that spreads fastest and lasts longest”. Around the world, one out of 10 adults dies because of smoking, with a number of deaths that is estimated to reach 6 million people every year: more than 600, 000 deaths are caused by passive smoking, while 5 million are due to active smoking (Bakan & Erci, 2018).

Tobacco use provokes a large number of morbidity and public health problems. As a matter of fact, it is a high-risk factor for a lot of pathologies and chronic preventable diseases, leading to a marked increase in health-care spending, such as lung cancer, asthma, chronic obstructive pulmonary diseases, coronary heart diseases, some types of vasculitis and stroke. These occur both in adult and in young population (Lee et al., 2018).

All around the world 30–40% of adults are smokers with an alarming increasing number of adolescents (Nuyts et al., in press). Data regarding health professionals are equally worrisome. According to an Italian cross-sectional study, 44% of 1082 Italian health-care workers are smokers: 33.9% of them are physicians, 49.8% nurses, 41.1% technicians, 50.4% auxiliary employees (Ficarra et al., 2010).

Health-care workers have a professional responsibility in advising and promoting smoking cessation, knowing all the risks linked to active and passive smoking (one of their primary aim would be prevention). They are seen as role models, so they should be an example in stopping smoking and supporting patients, struggling with them to reach this goal. However, although they recognize their prevention role, they continue to smoke (La Torre et al., 2011).

We tried to identify the best interventions to incentivize and promote smoking cessation in health-care workers through a search of studies in scientific literature. We have noticed that there is no common line of action in the interventions described in these studies and few trials have been published about this topic.

Therefore, we have decided to carry out a systematic review about smoking cessation interventions on health-care workers and a meta-analysis to clarify and to examine this issue and try to suggest the best intervention modality.

## Materials and Methods

This review was registered with PROSPERO: CRD42019130117. It was carried out on the basis of PICO strategy (Population: healthcare professionals; Intervention: smoking cessation intervention; Comparator: no intervention; Outcome: quitting smoking; Study design: all studies—clinical trials, cohort studies, cross-sectional studies).

### Search strategy

This systematic review was performed based on PRISMA statement (Liberati et al., 2009) and of Cochrane Handbook for Systematic Reviews of Intervention (Higgins & Thomas, 2019). The following databases were searched until December 2018: PubMed, Scopus, Web of Science and CINAHL. Articles were retrieved using the string “(Health care personnel OR hospital staff OR medical staff OR paramedical staff OR health care worker*) AND (smoking cessation OR smoking reduction OR stop smoking)”, which was adapted according to the research criteria of each database (we used the character “*” for PubMed and WOS database). We also reviewed the reference lists of the identified articles to avoid missing relevant studies (Wells et al., 2019).

### Inclusion and exclusion criteria

The following inclusion criteria were used: (1) randomized clinical trials (RCT); (2) cohort studies; (3) cross-sectional studies; (4) studies regarding health-care workers; (5) studies published in Italian, English, German or Spanish. Among included articles, there were quasi-experimental studies, i.e., research studies that resembles experimental research but is not true experimental research. Although the independent variable is manipulated, participants are not randomly assigned to conditions or orders of conditions (Cook & Campbell, 1979). The following exclusion criteria were used: (1) case-reports; (2) reviews; (3) case-control studies; (4) abstracts and author debates or editorials; (5) lack of effective statistical analysis; (6) animal studies; (7) *in vitro* studies; (8) studies dealing smoking cessation/reduction that did not include health-care workers as group of intervention.

No filter item about articles period of publication was applied.

### Data extraction

Following the inclusion criteria, four authors (VC, BD, AS and GT) independently selected the articles on the basis of relevant titles and abstracts. The full text of each selected paper was then evaluated to determine if it was suitable for inclusion. Disagreements were solved through consensus and by discussion with a reference author (GLT). For each eligible study, the following information was independently extracted by four authors (VC, BD, AS and GT) and examined by reference author (GLT): name of the first author, publication year, title, study design, country, type of intervention, number of health-care workers involved and results.

### Quality assessment

Quality of all studies included in the systematic review was assessed according to the Newcastle-Ottawa Scale (NOS) on cohort or cross-sectional studies (Wells et al., 2019) and to the Cochrane Risk of Bias Tool for Randomized Controlled Trials (Higgins & Thomas, 2019), when appropriate. Risk of bias for cohort or cross-sectional studies was performed by the Newcastle-Ottawa Scale (NOS) risk of bias assessment tool for observational studies that is recommended by the Cochrane Collaboration (Lo, Mertz & Loeb, 2014).

### Statistical analysis

Risk ratios were calculated for dichotomous outcomes. For meta-analysis, cohort studies and clinical trials were considered; in particular, we have considered cohort studies those with a quasi-experimental or a before-after design. The study from Sarna et al. (2009) was excluded from meta-analysis because it was not suitable for this procedure. Due to the heterogeneous nature of the papers included, we employed random effects models in all meta-analyses. Heterogeneity was documented by the *P* for homogeneity values reported in Results section. Subgroup analysis and meta-regression were based on fixed effects models. EpiSheet package was used for statistical analyses.

## Results

### Search results and characteristics of included studies for systematic review

**Figure 1:**
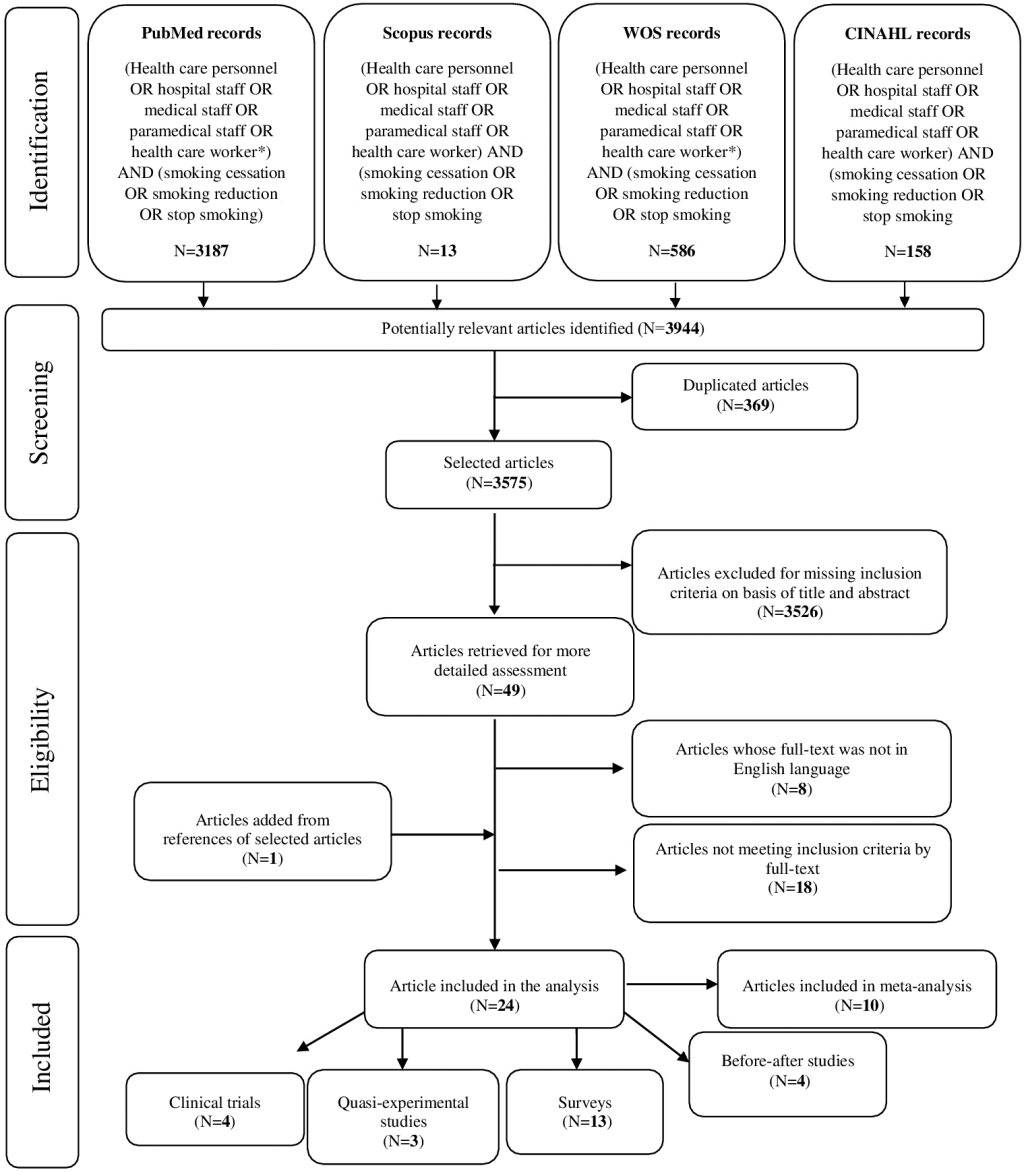
PRISMA flow diagram.

The search strategy gave a total of 3,944 references (3,187 in PubMed, 13 in Scopus, 586 in Web of Science and 158 in CINAHL): of those, 369 were duplicate references. The remaining 3,575 references underwent a title and abstract analysis, after which 3,526 were excluded. As a result, 49 articles were recovered for full-text reading: among them, 26 were excluded (8 did not comply with the language criteria of inclusion and their abstract reported no useful data; 18 did not meet inclusion criteria by full-text). One article was retrieved from snowball procedure and, finally, 24 articles have been included in the analysis (Fig. 1): there were 4 clinical trials (1 from UK, 1 from Croatia, 1 from Egypt and 1 from Switzerland), 3 quasi-experimental studies (2 from USA and 1 from UK), 4 before-after studies (2 from Spain, 1 from Turkey and the remaining 1 from Vietnam) and 13 surveys (2 from UK, 1 from Switzerland, 1 from Denmark, 1 from China, 2 from Australia, 2 from USA and 4 from Spain) (Table 1).

### Quality assessment

Quality assessment of before-after studies resulted in poor, fair and good quality in 1, 1 and 1 cases, respectively. All cross-sectional studies were of fair quality (ranging from 4 to 7 points), with the exception of two (Bloor, Meeson & Crome, 2006; Martínez et al., 2018), which had a poor quality: 3 points, both. Two quasi-experimental studies (Longo et al., 1996; Rowe & Clark, 1999) were of fair quality (ranging from 6 to 7 points) and the remaining one (Sarna et al., 2009) was of poor quality. Finally, all clinical trials were of fair quality, excluding one (Mohamed, Mustafa & Del Ghoneim, 2016), which was of poor quality (Table 1).

### Results for before-after studies

Results for before-after studies were varying (P for homogeneity <0.01) and depended on the type of intervention: they were positive except for one of them, which showed tiny post intervention differences (Table 1). At risk of bias analysis, three studies reached a good score: Battle et al. (1991) (6), Dao Thi Minh et al. (8) and Santinà et al. (6), whereas Bakan et al. obtained a poor score (4).

Dao Thi Minh et al. (2015) performed a pre- and post-intervention cross-sectional study in nine selected hospitals after a “smoke-free hospital model” implementation: smoking prevalence significantly decreased post-intervention, but the number of daily cigarettes smoked at workplaces among male health workers remained unchanged. Moreover, air nicotine levels in the doctors’ lounges and in emergency departments did not change post-intervention, while at other sites the levels decreased minimally. Finally, Santinà et al. (2011) assessed a plan entitled “Smoke-free hospitals” by conducting two surveys (in 2004 and in 2007): the number of smoking workers decreased significantly in all sectors, but was less marked in nursing staff. In 2007 people only smoked in smoking areas (*p* < 0.0001). The plan was supported by smokers and non-smokers.

### Results for cross-sectional studies

Cross-sectional studies were quite homogenous about the no smoking bans and policies implementation in hospitals; the smoking bans and policies introduction led to a smoking cessation or reduction among health-care workers (Table 1). At the risk of bias evaluation, two papers were scored 3 (Bloor, Meeson & Crome, 2006; Martínez et al., 2018), three were scored 4 (Jones, Crocker & Ruffin, 1998; Poder et al., 2012; Strobl & Latter, 1998), two scored 5 points (Agusti et al., 1991; Etter, Khan & Etter, 2008); among positive evaluations, four papers scored 6 (Martínez et al., 2012; Offord et al., 1992; Stillman, Hantula & Swank, 1994; Xiao et al., 2013) and two scored 7 points, because of the lowest risk of bias (ReyesUrueña et al., 2013; Kannegaard et al., 2005 ).

**Table 1:** Recap of results for included studies.

***Author, year. Country***	***Study design***	***Health care workers involved (n*^*o*^*)***	***Interventions***	***Main results***	***Quality assessment***
Battle, 1991. Spain.	Before-after study	599	Policy intervention: restrictions on smoking in hospital areas, with lectures on smoking and smoking cessation help.	Current smokers decreased from 51% to 40% and the ex-smokers increased from 16% to 23%.	Newcastle Ottawa for cohort studies: 6/8.
Augusti, 1991. Spain.	Cross-sectional study	211: 149 physicians and nurses (PS) and 62 other professionals without sanitary responsibilities (PNA).	Stop smoking program, combining group therapy, nicotine chewing gum and behavioral assistance through the evaluation of CO contained in expired breath.	30% quitted smoking. PS had a higher rate of quitting smoking than PNA (*p* < 0.05): 8% of PS and 1,6% of PNA stopped respectively.	Newcastle Ottawa for cross-sectional: 5/9.
Santinà, 2011. Spain.	Before-after study	2004 (*n* = 483); 2007 (*n* = 425)	Before-after “smoke-free plan” surveys to evaluate the prevalence of smokers in the hospital and the support of health workers for the plan.	Percentage of smoking workers decreased from 35.2% to 27.4% (*P* < .0.5), but was lower in nurses.	Newcastle Ottawa for cohort studies: 6/8.
Martinez, 2012. Spain.	Cross-sectional study	930	Implementation of the “Catalan Network of Smoke-free Hospitals” smoking cessation program for abstinence.	The abstinence probability was 0.504 after six months from the introduction of the program (95% CI [0.431–0.570]). It was higher in men (0.526, 95% CI [0.398–0.651]) than in women (0.495, 95% CI [0.410–0.581]) and physicians had better score (0.659, 95% CI [0.506–0.811]) than nurses (0.463, 95% CI [0.349–0.576]). The highest abstinence probabilities were recorded in hospital staff treated with nicotine replacement therapy plus bupropion (0.761, 95% CI [0.588–0.933]).	Newcastle Ottawa for cross-sectional: 6/9.
Reyes Uruena, 2013. Spain.	Cross-sectional study	2001 = 310; 2011 = 383.	A survey to examine the smoking habits among workers in two acute care Spanish institutions.	The final data showed the smoking prevalence among health care workers was 30.00% for 2001 and 29.42% for 2011. Smoking habits decreased in medical staff (from 25.97% in 2001 to 18.88% in 2011; *p* = 0.005) and in nurses (from 35.15% in 2001 to 25.61% in 2011; *p* = 0.007) but not among the administrative workers.	Newcastle Ottawa for cross-sectional: 7/9.
Dalsgareth, 2004. Denmark.	Clinical trial	336	The experimental group (*n* = 222) was treated with bupropion while control group (*n* = 114) with placebo. All participants were motivated to quit smoking and received behavioral counseling.	At week 7, 43% in the bupropion group and 18% in the placebo group (*p* < 0.001) were continues abstainers; after 26 weeks, 18% in bupropion group and 7% in placebo group (*p* = 0.008). Bupropion was effective as an aid to smoking cessation.	Cochrane Risk of Bias Tool for Randomized Controlled Trials: Fair Quality
Kannegaard, 2005. Denmark.	Cross-sectional study	1999 (*n* = 726); 2001 (*n* = 724)	A survey conducted in 2001 to compare the smoking habits and attitudes among hospital staff with results obtained in 1999.	The percentage of smokers significantly decreased from 33% to 26%. A small number of workers were less concerned by passive smoking in 2001. Two out of three respondents thought it was right implementing sanctions against the members of staff who broke the rules.	Newcastle Ottawa for cross-sectional: 7/9.
Zellweger, 2005. Switzerland.	Clinical trial	687	The participants were randomized in intervention group treated with Bupropion SR (*n* = 517) and in control group, treated with placebo (*n* = 107) for 7 weeks.	The 50% of intervention group and the 40% of control group reached the continuing abstinence on a 4-week period (*p* = 0.013). At week 7, nurses in Bupropion SR group had a higher abstinence rate than doctors (52% compared with 42%). This difference was observed also in placebo group (44% compared with 31%). After 52 weeks the 23% of the subjects in intervention group and the 22% in control group were abstinent. Also, the abstinence rates for nurses and doctors were similar (24% for nurses and 23% for doctors).	Cochrane Risk of Bias Tool for Randomized Controlled Trials: Fair Quality
Etter, 2008. Switzerland.	Cross-sectional study	2003 (*n* = 106); 2004 (*n* = 134).	Implementation of a partial smoking ban followed by a total smoking ban	More smokers were offered nicotine replacement products by hospital-staff after the ban implementation (from 13% to 52%, *p* < 0.001). 55% of the participants considered the total ban too strict and the 64% preferred the partial ban.	Newcastle Ottawa for cross-sectional: 5/9.
Glavas, 2003. Croatia.	Clinical trial	107	The participants were divided in 2 groups: intervention group (*n* = 54) applying daily a transdermal nicotine system (TNS) patch over 3 weeks and control group (*n* = 53) receiving placebo.	After the 3-week intervention period, abstinence rates were 39% in the TNS group and 20% in the control group (chi-square test, *p* = 0.038). After one year, the rates were 23% and 16% respectively (*p* = 0.476) and climbed down to 18% and 14% (*p* = 0.797) 5 years later.	Cochrane Risk of Bias Tool for Randomized Controlled Trials: Fair Quality
Bakan, 2018. Turkey.	Before-after study	63	Behavioral therapy: HBM and TTM model	15% of nurses in HBM and 7% in TTM passed in action stage.	Newcastle Ottawa for cohort studies: 4/8.
Strobl and Latter, 1998. UK.	Cross-sectional study	33	Implementation of a smoking ban in a British teaching hospital.	Reduction in work-time cigarette consumption was not statistically significant (Wilcoxon test: *p* = 0.069). Six (21.4%) smokers stated that the ban helped them to try quitting smoking and two of three former smokers reported that it helped them to stop. Twenty (76.9%) of current smokers indicated their wish to give up.	Newcastle Ottawa for cross-sectional: 4/9.
Rowe, 1999. UK.	Quasi experimental study	110	Observation and examination of the effectiveness of a smoking cessation intervention for nurses. The authors located the nurses in a comparison and in a control group (nurses who wish help to give up smoking).	The results show that the 24% of student and qualified nurses in the intervention groups stopped smoking compared with 7% of those in the comparison groups: the differences are statistically significant (Fisher’s Exact Probability Test p<0,05).	Newcastle Ottawa for cohort studies: 6/8.
Bloor, 2006. UK.	Cross-sectional study	92	Policy intervention on nursing staff.	The Trust policy was supported by 31.5% of the staff (53.6% of never smokers, 37.5% of former smokers, 6.3% of smokers). The 69.5% of respondents felt that the non-smoking policy was useless on staff (53.6% of never smokers, 68.8% of former smokers. The 84.8% of the staff felt that the policy was useless to them and only 6.3% disagreed.	Newcastle Ottawa for cross-sectional:3/9.
Offord, 1992. USA.	Cross-sectional study	Pre Survey=7039; Post Survey=10560.	Introduction of a “Smoke-Free Poli**c**y”.	The prevalence of cigarette smoked decreased (from 16.7% to 13.8%) and a smoking cessation rate of 22.5% among smokers was calculated (*n* = 1562). During the follow-up period, many smokers have been in the action stage of cessation (37.1% attempt to stop smoking, 20.7% had used nicotine therapy and 13.8% had attended a formal cessation program).	Newcastle Ottawa for cross-sectional: 6/9.
Stillman, 1994. USA.	Cross-sectional study	Pre intervention=1696; Post intervention=1071.	Implementation of a smoking ban in a USA hospital.	A decrease was found: 2.1% of the physicians (*p* < 0.03) and 11.7% (*p* < 0.0001) affirmed to be smokers. Both physicians (93.7%) and nurses (87%) were favorable for a smoke-free policy, but nurses were more accommodating toward smoking and less to enforce a ban. Physicians were more favorable than nurses to stop smoking.	Newcastle Ottawa for cross-sectional: 6/9.
Longo, 1996. USA.	Quasi experimental study	709	Observation of the effects of workplace smoking bans in an American hospital. Current or former smokers working in smoke-free hospitals represented the intervention group and current or former smokers employed in a non-smoke-free workplace (not a hospital) the comparison group.	One-year after the ban, the quit ratio was 0.066 for intervention group (95% CI [0.050–0.082]) and 0.038 for comparison group (95% CI [0.025–0.052]; *p* = 0.02). Five years later it was 0.506 and 0.377 respectively. Pre-contemplative stage: 31.9% of intervention group and 46.8% of comparison group. Contemplative stage: 30.5% of intervention group and 23.4% of comparison group. Maintenance stage: 21.2% of intervention group and 12.9% of comparison group.	Newcastle Ottawa for cohort studies: 6/8.
Sarna, 2009. USA.	Quasi experimental study	246	Evaluation of the “Nurses QuitNet” effectiveness, an Internet-based smoking cessation program to support nurses in quitting smoking.	Quit rates were of 43%, 45% and 53% after 3, 6 and 12 months respectively after the program introduction. Total time spent on the website was significantly higher for those who succeeded in quitting. Stop smoking was influenced by workplace factors.	Newcastle Ottawa for cohort studies: 3/8.
Martinez, 2018. Bolivia, Guatemala and Paraguay	Cross-sectional study	Pre=202; after six months=99.	Online training program to stop smoking. There was surveyed the pre-post performance of the 5A’s by hospital workers.	There was an increase in the performance of the 5A’s components (Ask from 7 to 9; Advise 7 to 9; Assess 6 to 8; Assist 2 to 7 and Arrange 0.52 to 5; all *p* < 0.001).	Newcastle Ottawa for cross-sectional: 3/9.
Mohamed, 2016. Egypt.	Clinical trial	150	Participants were assigned at 2 different groups: control group (Group I; *n* = 111) treated by behavioral therapy and intervention group (Group II; *n* = 39) treated by behavioral therapy plus pharmacotherapy (Bupropion SR tablet). Both the groups were treated for three months.	The successful cessation rate was 48% and the failed cessation rate was 52%. There was a statistically significant higher successful rate in group II (69.3%) than in group I (40.5%).	Cochrane Risk of Bias Tool for Randomized Controlled Trials: Poor Quality
Xiao, 2013. China.	Cross-sectional study	2009=24642; 2010=24087.	Implementation of a smoking ban in 41 hospitals across 20 Chinese provinces	The smoking prevalence among workers decreased from 14.8% to 10.7% (p <.001). The authors realized that worker’s education was the key priority to help to stop smoking.	Newcastle Ottawa for cross-sectional: 6/9.
Dao Thi Minh, 2015. Vietnam.	Before-after study	1776	Implementation of a “smoke-free hospital model”	The percentage of current smokers among health professionals decreased from 14.8% to 7.3%. The prevalence of male health workers smokers was much higher than that of female workers (from 35.2% to 1.1% for male and from 20.1% to 0.2% for female). There were not significant changes in the number of cigarettes smoked per day (8.7 versus 10.3) and that of those smoked at the workplace (4.0 versus 3.4).	Newcastle Ottawa for cohort studies: 8/8.
Jones, 1998. Australia.	Cross-sectional study	111 (21 were not contactable after three months)	Implementation of a “Stop Smoking Program” to encourage employees to quit smoking, offering nicotine patches and support on a weekly basis.	The 8.1% of the staff involved in the study quitted smoking after implementation of the program, while the 71.1% remained smokers.	Newcastle Ottawa for cross-sectional: 4/9.
Poder, 2012. Australia.	Cross-sectional study	599	Introduction of “Sydney South West Area Health Service’s Smoke- free Environment Policy”.	There was a 61% reduction of observed smoking incidents 2 weeks after implementation, 46% at 6 months, 41% at 12 months, 51% reduction at18 months and 36% at 2 years after implementation (*p* ≤ 0.05). There was an overall reduction also in staff (44%; *p* ≤ 0.05) and in visitors (37%; *p* ≤ 0.05).	Newcastle Ottawa for cross-sectional: 4/9.

Martínez et al. (2012) found that significant predictors of abstinence were smoking 10 to 19 cigarettes/day, having present low or medium Fagerström Test for Nicotine Dependence score, and using combined treatment (nicotine replacement therapy and bupropion). The study of Offord et al. (1992) obtained a significant contribution toward providing a healthful work environment and toward encouraging non-smoking behavior in staff and patients by implementing a smoke-free policy in their medical center: decrease in prevalence was the result of both smoking cessation among existing employees and less frequent regular smoking among new employees. ReyesUrueña et al. (2013) in their study demonstrated that tobacco consumption reduction coincided with measures introduced by legislative changes, even if in different manner among health-care workers. Also Stillman, Hantula & Swank (1994) found that physicians and nurses differed significantly on attitudes related to implementation and enforcement of a smoking ban (nurses were more accommodating toward smoking and less to enforce a ban). Strobl & Latter (1998) suggested that smoking policies should aim at strengthening nurses’ determination to give up as well as secure their support for the restrictions in order to assist them in changing their smoking behaviour. Xiao and his coworkers (Xiao et al., 2013) realized that health-care workers’ education was the key priority to stop smoking.

### Results for quasi-experimental studies

Three studies (Longo et al., 1996; Rowe & Clark, 1999; Sarna et al., 2009) were considered quasi-experimental studies because they provided no randomization of participants. Quasi-experimental studies analyzed the effects of stop smoking interventions on health-care workers, with one exception (the authors analyzed the effect of a smoking bans). The total results, reported below, are favorable and satisfying (Table 1). The risk of bias analysis showed that two studies (Longo et al., 1996; Rowe & Clark, 1999) reached a good score (6), whereas the study from Sarna et al. obtained a poor score (3).

Longo et al. (1996) found that workplace smoking bans could be effective in saving lives, reducing health care costs, addressing safety concerns, and decreasing operating and maintenance expenses of employers. Rowe & Clark (1999) supported the effectiveness of offering an individualized approach utilizing also salivary cotinine measurements of continuous abstinence at 6 months and 1 year and demonstrated that 24% of student and qualified nurses in the intervention groups stopped smoking compared with 7% of those in the comparison groups, reaching statistical significance. Sarna et al. (2009) demonstrated that use of Nurses QuitNet and workplace factors influenced the nurses to stop smoking.

### Results for clinical trials

Since the trial studies designs were very similar, the authors wanted to search the most effective method to help health care professionals to quit smoking (*P* for homogeneity: 0.061). We could find two different groups of subjects included for each study: one undergoing pharmacotherapy (bupropion or nicotine) and the other one undergoing behavioral therapy or placebo. The results were positive and substantial for all studies, with the experimental group (the one traded with pharmacotherapy) having grater results in quitting smoking than control group (Table 1).

Dalsgareth et al. (2004) demonstrated that bupropion was effective as an aid to smoking cessation in a broad group of hospital employees in Denmark. Glavas, Rumboldt & Rumboldt (2003) proved that short-term transdermal nicotine system was effective in smoking cessation. Mohamed, Mustafa & Del Ghoneim (2016) found that programs promoting smoking cessation including behavioral therapy in addition to the complementary role of pharmacotherapy with bupropion enhanced the chance of success in smoking cessation. Finally, Zellweger et al. (2005) found that bupropion was effective and well tolerated in health care professionals, but prevention measures for relapse were needed to maintain long-term tobacco abstinence among healthcare workers.

### Meta-analysis results

For meta-analysis procedure, we have considered cohort studies and clinical trials. We have excluded cross-sectional studies since their study-design can cause biases in the result of our study. We have considered as cohort studies quasi-experimental and before-after studies design.

**Figure 2:**
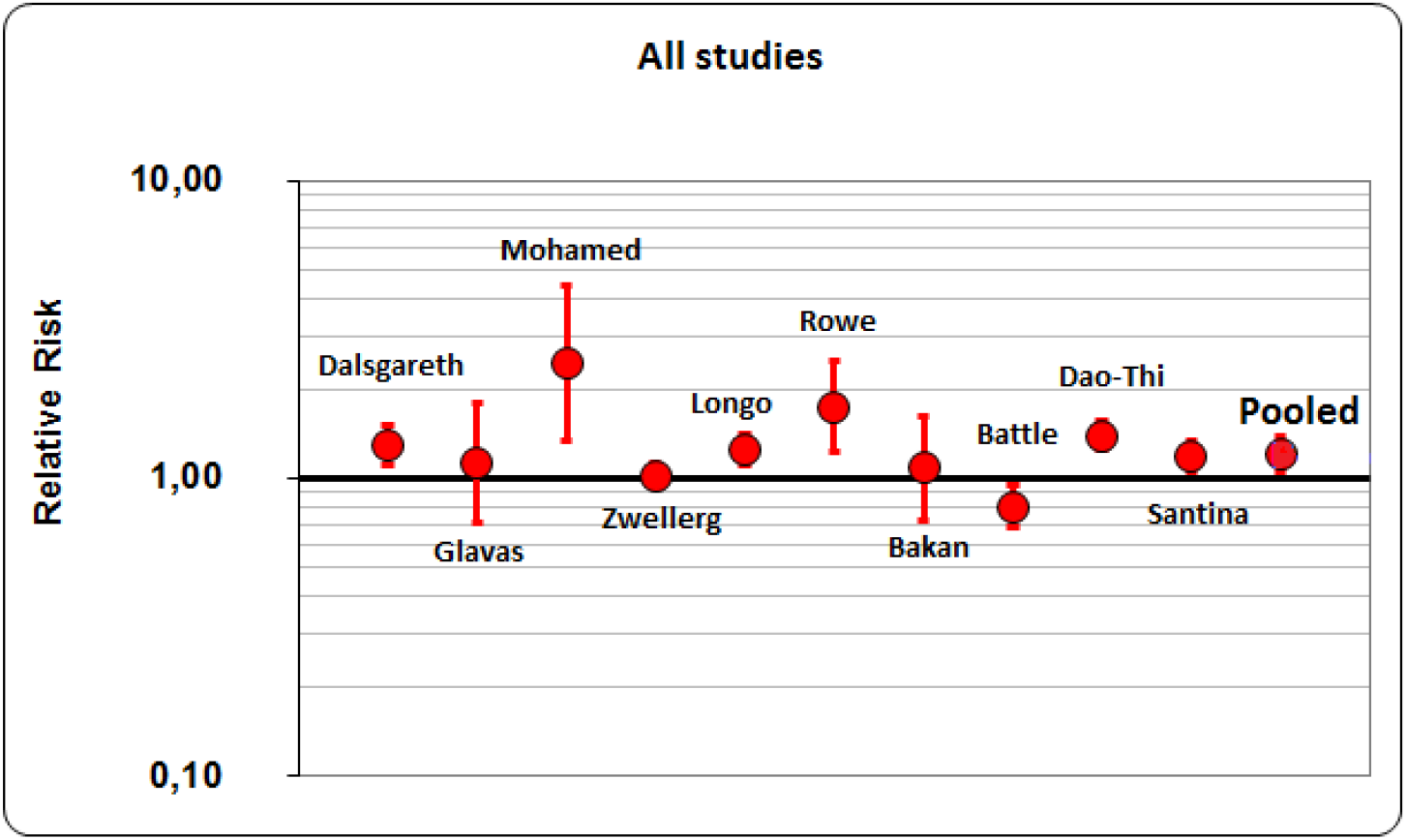
Pooled RR (Risk ratio)of all studies.

Overall, we have performed meta-analysis on 10 studies (6 cohort studies and 4 clinical trials; *P* for homogeneity <0.01). All the considered studies just reached the limit of statistical significance. A 21% of success rate resulted from the application of smoking cessation interventions, pharmacological or behavioral. The resulted pooled RR (Risk ratio) was 1.21 (95% CI [1.06–1.38]) (Fig. 2). Then, we have considered and analyzed the studies on the basis of their design: a 24% of success rates resulted from clinical trials (pooled RR was 1.244; 95% CI [1.099–1.407]) and 19% from cohort studies (pooled RR was 1.192; 95% CI [0.996–1.426]). Considering studies that reached almost a fair quality score (*n* = 8) to reduce variability and to ensure the good quality of our study (i.e., fair quality by Cochrane scores, ≥6/9 for cohort studies by Newcastle-Ottawa scale), the rate of success reached 18%, being 10% for 3 clinical trials and 21% for 5 cohort studies. The pooled RR for all studies was 1.179 (95% CI [1.030–1.350]), the one for clinical trial was 1.099 (95% CI [1.011–1.194]) and for cohort studies it was 1.206 (95% CI [0.993–1.465]). As a result, after this additional analysis, success rate decreased by three points for all studies (from 21% to 18%) and fourteen points for clinical trials (from 24% to 10%), but a little increase was registered for cohort studies (from 19% to 21%). Considering Clinical Trials, two studies (Glavas, Rumboldt & Rumboldt, 2003; Zellweger et al., 2005) have confidence intervals which include unity and one study (Mohamed, Mustafa & Del Ghoneim, 2016) has a wide confidence interval; as a consequence, the meta-analysis for its results depends heavily on one single study (Dalsgareth et al., 2004).

Meta-regression analysis was completed in order to analyze the influences of the quality, design and size of the studies on our study: we have found that if we weighted studies for the number of participants, the higher the quality of the study, the lower the relative risk was. As a result, the quality of the study may interfere with its result (*R*^2^ =  − 0.365).

## Discussion

Our revision took into account 24 articles retrieved from medical literature and published between 1991 and 2018. Spain was the most represented country in our analysis, followed by USA and UK. No studies in Italy were found. Overall, the majority of the retrieved articles reported positive results in terms of percentage of health-care workers that stopped smoking habit: in particular, excluding one before-after study, all articles of the same design reported positive results; among cross-sectional studies, 10 obtained a gain in health-care workers that passed to action, one did not show changes and the remaining two did not reach the aim to increase the proportion of people stopping their habit (to notice the cross-sectional results about nurses, showing worst cessation scores than physicians and about women workers, showing less smoking cessation percentage than their male colleagues); among clinical trials, all of them reported positive results in terms of smoking cessation of health-care workers, with a preference for pharmacological approaches respect to placebo or behavior therapy; among quasi-experimental studies, all of them reported positive results. If we considered statistically significant results, 15 studies reached this goal and only 1 reported borderline significant results; the remaining studies with positive results did not reach statistical significance.

Overall, meta-analysis showed about 1/5 probability of success in getting rid of smoking. If we tried to divide the studies on the basis of their designs, clinical trials obtained a greater probability of success than cohort studies (about 1/4 vs 1/5) and this result was atypical. We believe that these findings were due to the fact that health-care workers involved in clinical trials are more motivated to stop smoking and pass to action than in other study designs, such as those reporting policies. However, two studies (Glavas, Rumboldt & Rumboldt, 2003; Zellweger et al., 2005) have confidence intervals which include unity and one study (Mohamed, Mustafa & Del Ghoneim, 2016) has a wide confidence interval; as a consequence, the meta-analysis for its results depends heavily on one single study (Dalsgareth et al., 2004). We have carried-out also meta-regression analysis in order to find if the results of our study were influenced by the quality, design and size of the studies: we have found that if we weighted studies for the number of participants, the higher the quality of the study, the lower the relative risk was.

The integration of behavioral health services in primary care settings presents an opportunity to enhance the delivery of tobacco cessation interventions in the primary care setting (Wray et al., 2018). Wray et al. (2018) performed a meta-analysis which summarized the outcomes of brief behavioral interventions targeting tobacco use that can be delivered in integrated primary care (IPC) settings. In their study, patients in the intervention groups exhibited significantly greater odds of smoking cessation compared with those in the comparison groups (OR = 1.78, *p* < .001). Subgroup analyses did not reveal significant sources of heterogeneity attributable to moderators such as methodological quality, gender, bioverification, follow-up time period, or intervention characteristics (such as setting, type, or length of intervention). As a consequence, brief tobacco cessation interventions delivered in IPC settings demonstrated to be effective. The integration of behavioral health services into primary care represented an opportunity to increase the delivery of tobacco cessation interventions, as behavioral health providers in these settings were experts in behavior change interventions and may have more time to deliver these interventions than primary care providers (Wray et al., 2018).

The strength of our study lies in the fact that we have systematically reviewed all scientific literature on this topic, considering different kind of studies that evaluated different types of interventions; additionally, we tried to compare the results of different studies considering confounding variables and performing analysis in a separate manner, to elicit the role of eventual confounding variables. While the heterogeneity of the populations is a constant issue in a meta-analytic approach, we considered as an intervention all types of interventions (pharmacological and/or behavioural) and outcome measure as the smoking cessation. This approach, according to us, is sufficient to justify a meta-analysis. We have also considered works from European countries with different legal regulations and with their own health and social context: some of these points could contribute to the heterogeneity of the studies considered and to the heterogeneous results, but on the other hand we have shown a comprehensive and exhaustive analysis for determining what may be the best approach in a particular social and health context. Meta-analysis data were performed considering all studies and also considering studies on the basis of their own design: this choice permitted to confirm if other variables determined the results of our study.

Limitations of our study must be acknowledged. First of all, we could have introduced a bias making a pooled analysis considering all the study design. However, this was justified by the low number of papers retrieved. Moreover, the majority of the papers included in the review dealt with cross-sectional studies and these ones are not the best option to evaluate the results of a smoking cessation intervention; we found few experimental studies, which also considered a limited number of people. However, excluding 5 studies of poor quality, the majority of retrieved papers were of fair/good quality, which gave support to the results of our work. Another limitation ire related to the presence of heterogeneity of the results between studies, but this could be partly solved to the use of a random effect model in the meta-analysis that took into account the variability between studies.

In a public health perspective, the role of a healthcare worker is important, not only because he/she has a role in prescribing or suggesting the best treatment options in a patient asking help in stopping smoking, but also because he/she represents an example for people regarding their lifestyle and a smoking healthcare worker certainly does not motivate other people to stop smoking. Giving their dual role of model and therapeutic guide, it is better that health-care workers try to stop smoking for motivating efficaciously people to stop smoking; namely, one of their main roles is to promote healthy lifestyles and guiding people to adopt good behavioral strategies for improving quality of life. Consequently, smoking cessation intervention oriented on health-care workers can permit to obtain better results also in a public health perspective. More attention is needed for nurses and female health-care workers, that showed a bigger difficulty and failure in stop smoking, probably linked to a higher stress due to a greater workload for nurses and to their social status for women HCWs.

## Conclusions

Health professionals are expected to play an active role in fighting against smoking habit and to be models for patients; they also have the power to influence patients they provide care in terms of health promotion for appropriate lifestyles. Our study demonstrated that either policies and pharmaceutical interventions can be able to obtain positive results in terms of reduction of the proportion of health-care workers with a smoking habit; about a 20% of success rate can result from the application of smoking cessation interventions, either pharmacological or behavioral. However, as shown by our review, combination approaches can produce better results in terms of cessation percentages and smoking abstinence.

##  Supplemental Information

10.7717/peerj.9396/supp-1 Supplemental Information 1 PRISMA checklist

10.7717/peerj.9396/supp-2 Supplemental Information 2 Systematic Review and/or Meta-Analysis Rationale

10.7717/peerj.9396/supp-3 Supplemental Information 3 All studies

10.7717/peerj.9396/supp-4 Supplemental Information 4 Cohort studies

10.7717/peerj.9396/supp-5 Supplemental Information 5 Only clinical trial

## Additional Information and Declarations

### Data Availability

The following information was supplied regarding data availability: Episheet files for the meta-analysis are available in the Supplemental Files.
