# Supplementary material for: Smoking cessation interventions on health-care workers: a systematic review and meta-analysis
Source: PeerJ. 2020 Jun 16;8:e9396. doi: 10.7717/peerj.9396 (PMC7304418; doi:10.7717/peerj.9396)
Supplement: Supplemental Information 2 [file peerj-08-9396-s002.docx]

**SUPPLEMENTAL FILE**

**Rationale for conducting meta-analysis**

The aim of our review was to enhance the readers’ knowledge about different types of stop smoking interventions on health care workers and to identify the best one; but to assess if they were really effective, we considered necessary a meta-analysis realization. It was not a standard meta-analysis, as a matter of fact it includes both clinical trials and cohort studies, but this ensemble was important to complete our results and to have a full vision of them (literature is poor in clinical trials regarding smoking cessation intervention, for this reason we decided to include also cohort studies).

**Meta-analysis contribution to knowledge**

We conducted six different meta-analysis: one regarding 4 clinical trials and 6 cohort studies, one only clinical trials, one only cohort studies and the remaining three regarding the previously ones but including only studies of good quality to make sure possible biases deleting (3 clinical trials and 4 cohort studies). Results showed us that overall RR was > 1 for all meta-analysis, meaning that all interventions reported in the studies were effective. They were all statistically significant, except for cohort studies. Furthermore, we noticed that meta-analysis results for clinical trials were more favourable than results for cohort studies and this was unexpected, maybe linked to the fact that healthcare workers involved in clinical trials were more motivated to stop smoking and pass to action than in other study designs. We think that declaring this results to readers and to other health care workers might improve their knowledge about the health care intervention effectiveness. Comparing our results with other meta-analysis was complicated because we did not find them in our research and we noticed also that literature is poor in meta-analysis regarding this topic. It is also true that there are few trials in literature and realizing a meta-analysis could be considered complex. Despite this, we made it and this is an added value to our research.

We also completed a meta-regression analysis to support the study objectivity. We examined the influence of the quality, design and size of the studies included in our study: we have found that waiting the studies on the number of participants, the higher the quality the lower the lower the relative risk was, so the quality of the study might interfere with its result (R^2^=-0.365).

Evaluating other literature reviews, they included interventions just regarding certain health care workers, like nurses or physicians. Our review, instead, took in consideration all health care workers giving, as a consequence, a whole vision of the state of the art.
